# A Multivalent and Cross-Protective Vaccine Strategy against Arenaviruses Associated with Human Disease

**DOI:** 10.1371/journal.ppat.1000695

**Published:** 2009-12-18

**Authors:** Maya F. Kotturi, Jason Botten, John Sidney, Huynh-Hoa Bui, Lori Giancola, Matt Maybeno, Josie Babin, Carla Oseroff, Valerie Pasquetto, Jason A. Greenbaum, Bjoern Peters, Joey Ting, Danh Do, Lo Vang, Jeff Alexander, Howard Grey, Michael J. Buchmeier, Alessandro Sette

**Affiliations:** 1 Division of Vaccine Discovery, La Jolla Institute for Allergy and Immunology, La Jolla, California, United States of America; 2 Vermont Center for Immunology and Infectious Diseases, The University of Vermont College of Medicine, Burlington, Vermont, United States of America; 3 Departments of Molecular Biology and Biochemistry and Community and Environmental Medicine, University of California, Irvine, California, United States of America; 4 Pharmexa-Epimmune, San Diego, California, United States of America; Mount Sinai School of Medicine, United States of America

## Abstract

Arenaviruses are the causative pathogens of severe hemorrhagic fever and aseptic meningitis in humans, for which no licensed vaccines are currently available. Pathogen heterogeneity within the *Arenaviridae* family poses a significant challenge for vaccine development. The main hypothesis we tested in the present study was whether it is possible to design a universal vaccine strategy capable of inducing simultaneous HLA-restricted CD8^+^ T cell responses against 7 pathogenic arenaviruses (including the lymphocytic choriomeningitis, Lassa, Guanarito, Junin, Machupo, Sabia, and Whitewater Arroyo viruses), either through the identification of widely conserved epitopes, or by the identification of a collection of epitopes derived from multiple arenavirus species. By inoculating HLA transgenic mice with a panel of recombinant vaccinia viruses (rVACVs) expressing the different arenavirus proteins, we identified 10 HLA-A02 and 10 HLA-A03-restricted epitopes that are naturally processed in human antigen-presenting cells. For some of these epitopes we were able to demonstrate cross-reactive CD8^+^ T cell responses, further increasing the coverage afforded by the epitope set against each different arenavirus species. Importantly, we showed that immunization of HLA transgenic mice with an epitope cocktail generated simultaneous CD8^+^ T cell responses against all 7 arenaviruses, and protected mice against challenge with rVACVs expressing either Old or New World arenavirus glycoproteins. In conclusion, the set of identified epitopes allows broad, non-ethnically biased coverage of all 7 viral species targeted by our studies.

## Author Summary

Arenaviruses cause significant morbidity and mortality worldwide and are also regarded as a potential bioterrorist threat. CD8^+^ T cells restricted by class I MHC molecules clearly play a protective role in murine models of arenavirus infection, yet little is known about the epitopes recognized in the context of human class I MHC (HLA). Here, we defined 20 CD8^+^ T cell epitopes restricted by HLA class I molecules, derived from 7 different species of arenaviruses associated with human disease. To accomplish this task, we utilized epitope predictions, *in vitro* HLA binding assays, and HLA transgenic mice inoculated with recombinant vaccinia viruses (rVACV) expressing arenavirus antigens. Because our analysis targeted two of the most common HLA types worldwide, we project that the CD8^+^ T cell epitope set provides broad coverage against diverse ethnic groups within the human population. Furthermore, we show that immunization with a cocktail of these epitopes protects HLA transgenic mice from challenge with rVACV expressing antigens from different arenavirus species. Our findings suggest that a cell-mediated vaccine strategy might be able to protect against infection mediated by multiple arenavirus species.

## Introduction

Pathogen heterogeneity is a commonly encountered challenge for vaccine design. A considerable fraction of unmet vaccine needs for infectious disease is associated with pathogens naturally displaying significant levels of genetic diversity. In particular, RNA viruses pose a substantial challenge because of their propensity for rapid mutation and recombination. A prominent example includes arenaviruses, which are categorized in different complexes with distinct geographical locations, and continue to provide a formidable challenge for vaccine development.

The *Arenaviridae* family, consisting of a single genus (*Arenavirus*), contains 22 known viral species, which are classified phylogenetically into Old World and New World complexes [1]. The latter complex is further divided into three Clades (A, B, and C). Old World viruses include Lassa virus (LASV) and the prototypic arenavirus family member, lymphocytic choriomeningitis virus (LCMV). The New World Clade B contains some of the most pathogenic agents, including Guanarito virus (GTOV), Junin virus (JUNV), Machupo virus (MACV), Sabia virus (SABV), and the recombinant Clade A/B arenavirus, Whitewater Arroyo virus (WWAV). Arenaviruses are enveloped viruses, with a ∼10.7 kb RNA genome that encodes four viral proteins, including the glycoprotein precursor (GPC), nucleocapsid protein (NP), RNA-dependent RNA polymerase (L), and the zinc-finger binding protein (Z) [2].

Human infection with arenaviruses typically occurs through direct contact with infected rodents or by inhalation of infectious rodent excretions and secretions, and is associated with dehabilitating and sometimes life-threatening human disease, including central nervous system damage, aseptic meningitis [3], congenital deformities [4],[5], and severe hemorrhagic fever syndrome (reviewed in [6]). Despite the pathogenicity of arenaviruses, there are no licensed vaccines available. Therefore, it is important to develop novel prophylactic vaccine strategies to combat these viruses. CD8^+^ T cell responses have clearly been associated with reduced pathology and protection against Old World arenavirus infection in both murine models of infection [7],[8],[9],[10] and in humans [11],[12]. Previous reports also suggest a beneficial role for CD8^+^ T cell-mediated immunity in countering New World arenavirus infections in humans [13],[14]. Thus, vaccination strategies aimed at generating CD8^+^ T cell responses against both Old and New World arenaviruses should be considered.

The goals of the present study were to identify HLA-restricted CD8^+^ T cell epitopes from 7 different arenaviruses associated with disease in humans, including GTOV, JUNV, LASV, LCMV, MACV, SABV, and WWAV, and to develop a universal vaccination strategy designed to elicit a T cell-mediated immune response that would provide broad coverage against a variety of arenaviruses and across different ethnic populations. Because of the heterogeneity observed amongst different species of arenaviruses, two non-mutually exclusive concepts were explored. First, we wanted to address whether it was possible to identify CD8^+^ T cell epitopes that are conserved and/or cross-reactive amongst the different arenavirus species, and second, to determine whether it was possible to combine in the same vaccine, epitopes derived from each of the different arenavirus species, thus providing effective multivalent protection. Here, we report the identification of HLA-restricted CD8^+^ T cell epitopes that were either cross-reactive or species-specific, and demonstrate that immunization with these epitopes protected HLA transgenic mice from challenge with rVACV expressing antigens from different arenavirus species.

## Results

### Generation of a set of rVACV expressing arenavirus antigens

Because the majority of arenaviruses studied require biosafety level-4 (BSL-4) containment, rVACV constructs were designed to express the GPC, NP, L, or Z proteins from the 7 different arenaviruses as a tool to induce and evaluate CD8^+^ T cell responses. The gene sequences encoding the different arenavirus proteins were derived from prototypic virus strains (Table S1), and the constructs were engineered into the Western Reserve (WR) strain of VACV.

In total, individual rVACV constructs expressing 24 of the 28 arenavirus antigens of interest were generated. Arenavirus protein expression was confirmed for each rVACV through Western blot analysis with infected cell lysates. We verified that arenavirus proteins of the correct size were reproducibly detected from each construct (Figure S1 and data not shown). Although there were different levels of expression for the GPC, L, NP, and Z proteins within the same virus (as shown for GTOV in Figure S1), there was relatively little variation in the level of expression of the same viral protein across the 7 different arenavirus species. Protein expression remained relatively consistent across similar viral proteins as the large majority of arenavirus genes were under the control of a synthetic early/late promoter, PSYN. Overall, these results demonstrate that the 4 viral proteins from different arenavirus species can be ectopically expressed within a rVACV. We did not express WWAV L or Z protein because, at the initiation of this study, sequences for these viral antigens were not available. Technical difficulties also prevented the generation of JUNV Z and SABV L constructs.

### Bioinformatic selection of candidate arenavirus-derived CD8^+^ T cell epitopes

To identify candidate arenavirus-derived CD8^+^ T cell epitopes, we screened the GPC, L, NP, and Z protein sequences from GTOV, JUNV, LASV, LCMV, MACV, SABV, and WWAV using bioinformatic algorithms described elsewhere [15]. The HLA-A02 and HLA-A03 supertype specificities were selected because together they should allow coverage of ∼75% of the human worldwide population [16], and because HLA transgenic mice are available for these two HLA specificities [17],[18]. Because high HLA binding affinity is associated with immunogenicity *in vivo* [19], peptides with a predicted affinity of ≤100 nM were selected. To limit the number of candidates to a manageable number we chose to study up to 30 different peptides for each HLA/virus/antigen combination. In total, 481 HLA-A02 and 527 HLA-A03 unique candidate peptide sequences, consisting of nonamers and decamers, were selected and synthesized.

### Identification of HLA-A02-restricted arenavirus CD8^+^ T cell epitopes

**Figure 1:**
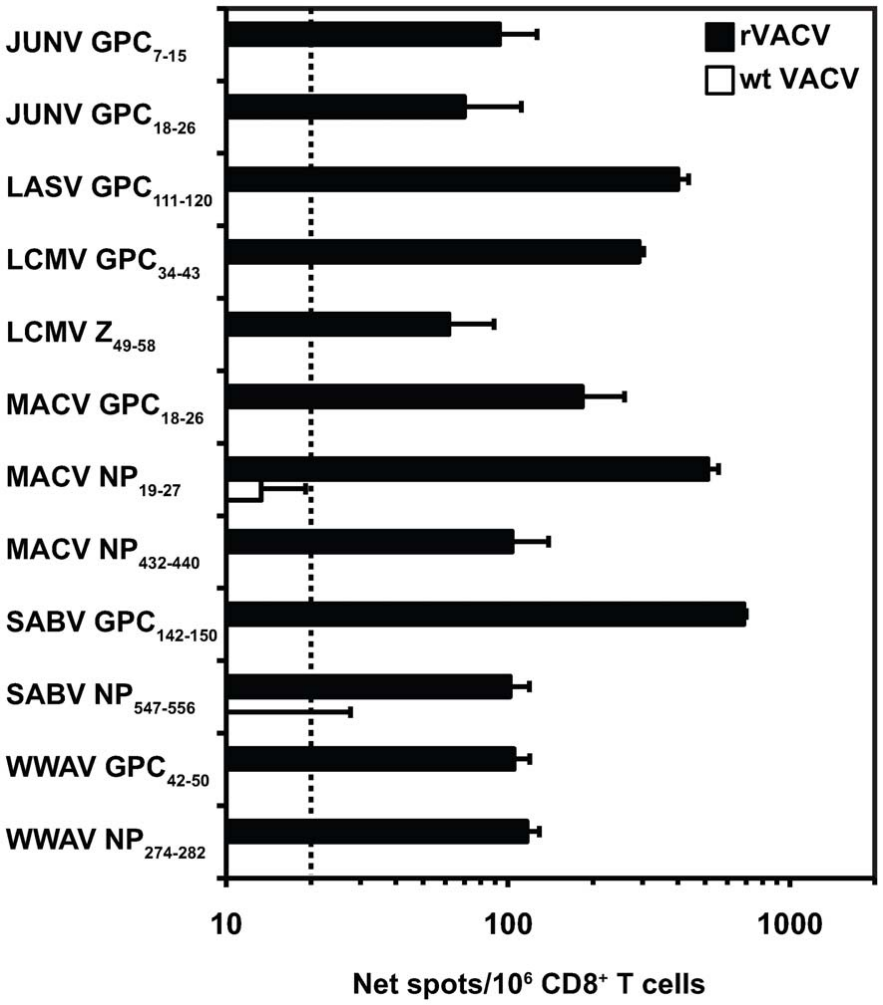
Identification of antigenic arenavirus-derived peptides in HLA-A*0201 transgenic mice following rVACV infection. HLA-A*0201 transgenic mice were immunized i.p. with either a rVACV expressing an arenavirus protein or wt VACV-WR as a control. Purified splenic CD8^+^ T cells were isolated 7 days later and exposed to human JA2.1 target cells, expressing the HLA-A*0201/K^b^ chimeric gene, that had been pulsed with 0.1 µg/ml of each of the listed peptides in an *ex vivo* IFN-γ ELISPOT assay. The dotted line indicates the threshold for peptide positivity (net SFC/10^6^ cells ≥20). Representative responses of at least two independent experiments are shown. Error bars indicate SD.

Because human PBMC samples from arenavirus-exposed individuals were difficult to obtain, we used HLA transgenic mice to identify human arenavirus epitopes. To determine the *in vivo* antigenicity of the 481 arenavirus peptides predicted to bind HLA-A02, HLA-A*0201 transgenic mice were infected with rVACV expressing one of the arenavirus proteins (see Materials and Methods for details). Twelve different CD8^+^ T cell epitopes from 6 arenavirus species were identified utilizing IFN-γ ELISPOT assays: 2 from JUNV (GPC_7–15_ and GPC_18–26_), 1 from LASV (GPC_111–120_), 2 from LCMV (GPC_34–43_ and Z_49–58_), 3 from MACV (GPC_18–26_, NP_19–27_, and NP_432–440_), 2 from SABV (GPC_142–150_ and NP_547–556_), and 2 from WWAV (GPC_42–50_ and NP_274–282_) (Figure 1). The JUNV GPC_18–26_ epitope is 100% conserved in MACV, and was independently identified as an epitope in both viruses. A CD8^+^ T cell epitope was not identified from GTOV. None of the epitopes were recognized by CD8^+^ T cells derived HLA-A*0201 transgenic mice infected with wild type (wt) VACV-WR.

**Figure 2:**
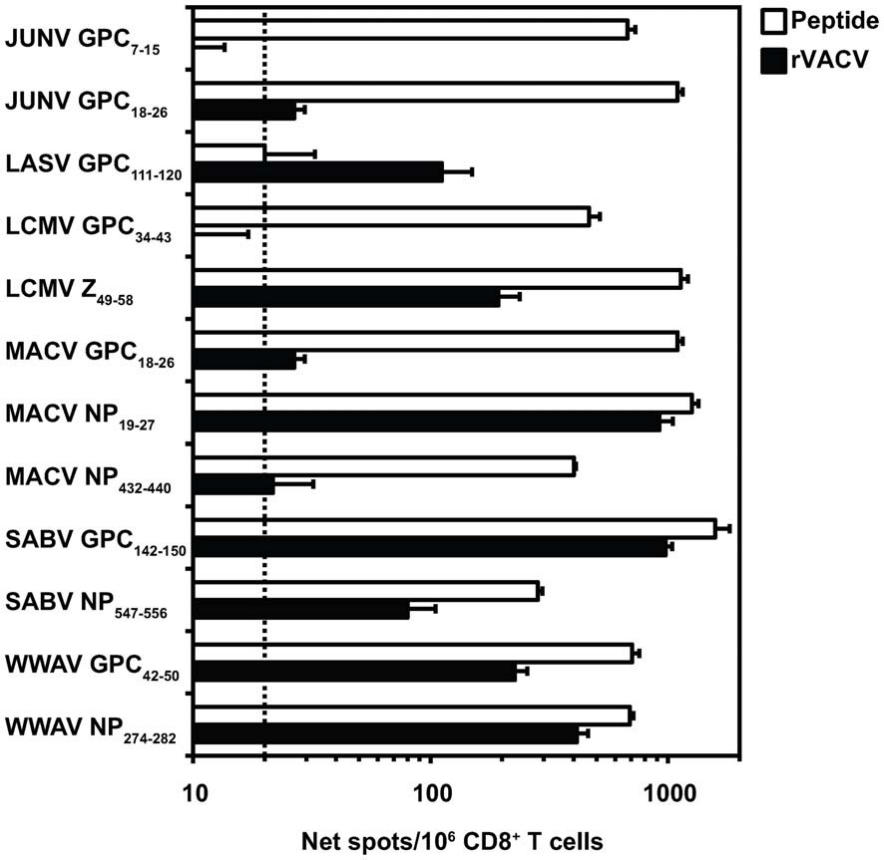
Natural processing of arenavirus peptides in HLA-A*0201-restricted human target cells that express native arenavirus antigens. HLA-A*0201 transgenic mice were immunized s.c. with one of the listed peptides, and 11 to 14 days later splenic CD8^+^ T cells were isolated. Effector CD8^+^ T cells were exposed to human JA2.1 cells that had been either pulsed with 1 µg/ml of the immunizing peptide, or infected with the appropriate rVACV in an *ex vivo* IFN-γ ELISPOT assay. The dotted line indicates the threshold for peptide positivity (net SFC/10^6^ cells ≥20). Error bars indicate SD. Data are representative of at least two independent experiments.

As these 12 HLA-A*0201 epitopes were identified following rVACV infection in transgenic mice, these experiments indicated that they can be generated by processing in mouse antigen-presenting cells (APCs). To assess processing in human APCs, HLA-A*0201 transgenic mice were immunized with the 12 arenavirus epitopes individually, and 11 to 14 days post-immunization, splenic CD8^+^ T cells were assayed for recognition of human target cells infected with the rVACV expressing the appropriate arenavirus antigen. Recognition of rVACV-infected target cells was robust for 7 of the 12 peptide-primed CD8^+^ T cells (LASV GPC_111–120_, LCMV Z_49–58_, MACV NP_19–27_, SABV GPC_142–150_, SABV NP_547–556_, WWAV GPC_42–50_, and WWAV NP_274–282_; Figure 2). Three epitopes (JUNV GPC_18–26_, MACV GPC_18–26_, and MACV NP_432–440_) were weakly recognized in replicate experiments, while for JUNV GPC_7–15_ and LCMV GPC_34–43_ processing in human APCs could not be demonstrated. None of the arenavirus peptide-primed CD8^+^ T cells had a detectable response to target cells infected with wt VACV-WR (data not shown). We also observed minor recognition of 2 epitopes by murine H-2^bxd^ as LASV GPC_111–120_ and LCMV GPC_34–43_ were presented by one of the endogenous mouse MHC class I molecules co-expressed in the HLA-A*0201 transgenic mice (data not shown). Table 1 provides an overall summary of the properties of the arenavirus-derived HLA-A*0201-restricted CD8^+^ T cell epitopes, including which epitopes were endogenously processed in human APCs.

**Table 1:** Properties of arenavirus-derived HLA-A*0201-restricted CD8^+^ T cell epitopes.

Arenavirus epitope^a^	Peptide sequence	% Identity to parental species^b^	Natural processing in human APCs^c^	Binding affinity to A*0201 (nM)^d^
JUNV GPC_7–15_	FMQEIPTFL	100	−	**11**
JUNV GPC_18–26_	ALNIALVAV	100	+/−	**79**
LASV GPC_111–120_	SIINHKFCNL	96	+	2123
LCMV GPC_34–43_	AVYNFATCGI	98	−	**137**
LCMV Z_49–58_	YLCRHCLNLL	99	+	**3.9**
MACV GPC_18–26_	ALNIALVAV	100	+/−	**79**
MACV NP_19–27_	GLSQFTHTV	94	+	**18**
MACV NP_432–440_	AMPGVLSYV	100	+/−	**8.4**
SABV GPC_142–150_	GLLEWIFRA	100	+	**1.2**
SABV NP_547–556_	LLPDALLFTL	100	+	**77**
WWAV GPC_42–50_	GLLQFIVFL	100	+	**<0.05**
WWAV NP_274–282_	TVIKTLLEV	100	+	**276**

a Peptide position within prototypic strains of arenavirus GPC, NP, L and Z proteins.

b Average % amino acid identity of epitope sequence to all full-length isolate sequences within a given arenavirus species.

c Each peptide was screened for its ability to be endogenously processed and presented by HLA-A*0201. Purified CD8^+^ T cells from peptide-immunized HLA-A*0201 mice were tested for recognition of human JA2.1 cells infected with the appropriate rVACV construct expressing one of the arenavirus proteins. +, Processed; +/−, weakly processed; −, not processed.

d Peptides were tested for binding to the HLA-A*0201 allele. Bold font highlights biologically relevant binders (IC_50_≤500 nM). −, peptides with binding affinity >5000 nM.

### Identification of HLA-A03-restricted arenavirus CD8^+^ T cell epitopes

Peptides containing positively charged amino acids at the carboxyl-termini associated with HLA-A03 supertype binding are not effectively processed in mice [20]. To overcome this potential problem, and enable the use of mice expressing the A03 supertype prototype allele HLA-A*1101, the immunogenicity of the 527 predicted HLA-A03 binding peptides was determined by immunization of HLA-A*1101 transgenic mice with pools of the predicted arenavirus peptides. Using this strategy, we identified 165 HLA-A03 supertype peptides that were immunogenic in HLA-A*1101 transgenic mice (data not shown).

**Figure 3:**
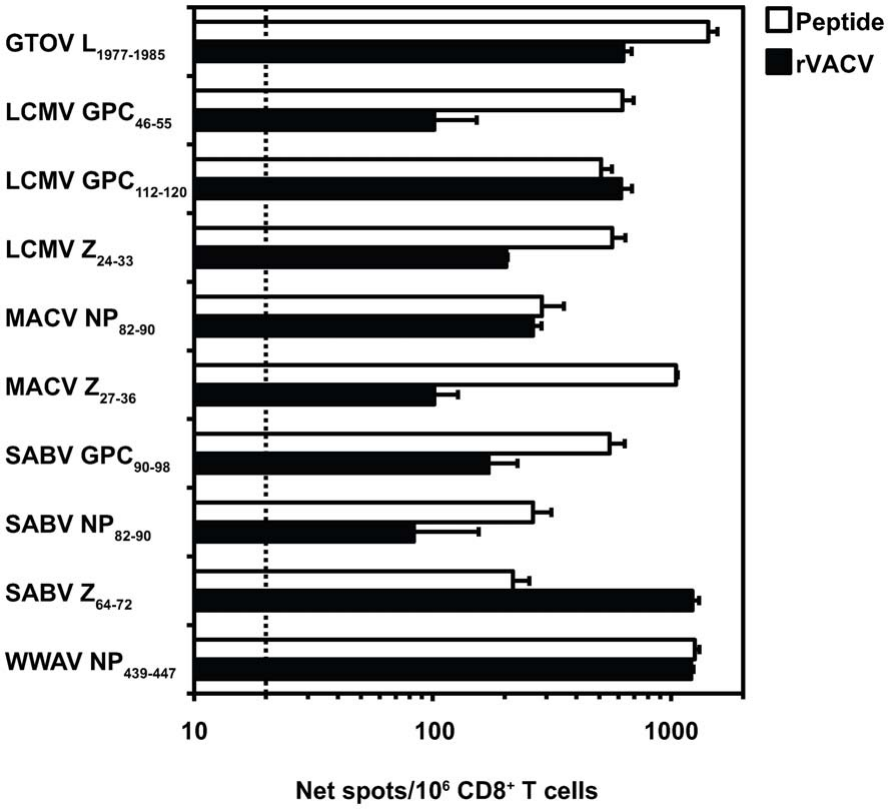
Identification of naturally processed arenavirus-derived peptides in HLA-A*1101 transgenic mice following peptide immunization. HLA-A*1101 transgenic mice were immunized s.c. with one of the listed peptides. After 11 to 14 days, splenocytes were restimulated for 6 days with the immunizing peptide, and CD8^+^ T cells were isolated. Effector CD8^+^ T cells were exposed to human BVR cells that had been either pulsed with 1 µg/ml of peptide, or infected with the appropriate rVACV in an *ex vivo* IFN-γ ELISPOT assay. The dotted line indicates the threshold for peptide positivity (net SFC/10^6^ cells ≥20). Error bars indicate SD. Results are representative of at least two independent experiments.

To assess processing of the immunogenic HLA-A*1101 peptides, HLA-A*1101 transgenic mice were immunized with individual arenavirus peptides (see Materials and Methods for details). At 11 to 14 days post-immunization, splenic CD8^+^ T cells were screened for recognition of HLA-A*1101-expressing human target cells that had been infected with an appropriate rVACV. It was found that 14 of the 165 HLA-A*1101 peptides tested were endogenously processed from native arenavirus antigens by human APCs and recognized by arenavirus peptide-primed CD8^+^ T cells. Of these peptides, 4 were overlapping with the 14 processed epitopes and had similar peptide-specific CD8^+^ T cell responses. Thus, in total, 10 unique epitopes were endogenously processed by human APCs. These peptides represented all 4 viral antigens and 5 different arenaviruses: 1 from GTOV (L_1977–1985_), 3 from LCMV (GPC_46–55_, GPC_112–120_ and Z_24–33_), 2 from MACV (NP_82–90_ and Z_27–36_), 3 from SABV (GPC_90–98_, NP_82–90_ and Z_64–72_), and 1 from WWAV (NP_439–447_) (Figure 3). A homologous epitope (NP_82–90_) was identified in both MACV and SABV. None of the arenavirus peptide-primed CD8^+^ T cells had a detectable response to target cells infected with wt VACV-WR (data not shown). In addition, a single determinant (LCMV GPC_46–55_) was immunogenic when presented by either HLA-A*1101-expressing or H-2^bxd^-expressing APCs (data not shown). An overall summary of the properties of the arenavirus-derived HLA-A*1101-restricted CD8^+^ T cell epitopes is provided in Table 2.

**Table 2:** Properties of arenavirus-derived HLA-A*1101-restricted CD8^+^ T cell epitopes.

Arenavirus epitope^a^	Peptide sequence	% Identity to parental species^b^	Natural processing in human APCs^c^	Binding affinity to A*1101 (nM)^d^
GTOV L_1977–1985_	ATVKNVVLR	100	+	**54**
LCMV GPC_46–55_	LVSFLLLAGR	89	+	**70**
LCMV GPC_112–120_	FTNDSIISH	89	+	**38**
LCMV Z_24–33_	TTYLGPLSCK	94	+	**2.8**
MACV NP_82–90_	SIQKNTIFK	97	+	**5.5**
MACV Z_27–36_	RTAPPSLYGR	97	+	**8.9**
SABV GPC_90–98_	STYYVHENK	100	+	**6.0**
SABV NP_82–90_	SSQRDTILK	100	+	**9.3**
SABV Z_64–72_	KCLNIMLGK	100	+	678
WWAV NP_439–447_	SSIIRSLPK	100	+	**2.5**

a Peptide position within prototypic strains of arenavirus GPC, NP, L and Z proteins.

b Average % amino acid identity of epitope sequence to all full-length isolate sequences within a given arenavirus species.

c Each peptide was screened for its ability to be endogenously processed and presented by HLA-A*1101. Purified CD8^+^ T cells from peptide-immunized HLA-A*1101 mice were tested for recognition of human BVR cells infected with the appropriate rVACV construct expressing one of the arenavirus proteins. +, Processed; −, not processed.

d Peptides were tested for binding to the HLA-A*1101 allele. Bold font highlights biologically relevant binders (IC_50_≤500 nM). −, peptides with binding affinity >5000 nM.

### Identification of HLA-restricted epitopes that are cross-reactive among multiple arenaviruses

The identified epitopes were 89 to 100% conserved within a given arenavirus species (Tables 1 and 2). Next, we wanted to determine whether the CD8^+^ T cell responses generated by the identified HLA-restricted epitopes could cross-reactively recognize orthologous sequences derived from different arenavirus species. Such cross-recognition might further increase the coverage afforded by the epitope set against each different arenavirus species.

To identify cross-reactive peptides, we compared the primary amino acid sequence of the 12 HLA-A02 epitopes, and the 10 HLA-A03 epitopes identified herein, as well as the 3 LASV (GPC_42–50_, GPC_60–68_, and GPC_441–449_) and 3 LCMV (GPC_10–18_, GPC_447–455_, and NP_69–77_) HLA-A*0201-restricted epitopes identified in previous studies [9],[10] to the orthologous regions within the other arenavirus protein sequences used to generate the rVACV constructs. Peptides containing 2 or more identical amino acids as the epitope sequence and found in ≥20% of the isolates in the arenavirus protein sequence database (http://epitope.liai.org:8080/projects/arena) were chosen for further analysis. Using this approach, 144 candidate cross-reactive peptides were selected.

To test cross-reactivity, HLA transgenic mice were immunized with individual arenavirus epitopes we had identified. Splenic CD8^+^ T cells were screened for IFN-γ secretion in response to APCs pulsed with either the immunizing epitope or the potentially cross-reactive arenavirus peptides. Immunization with 5 HLA-A*0201-restricted epitopes (JUNV GPC_18–26_, MACV GPC_18–26_, LCMV GPC_447–455_, MACV NP_19–27_, and MACV NP_432–440_) and 2 HLA-A*1101-restricted epitopes (LCMV GPC_46–55_ and MACV NP_82–90_) generated one or more cross-reactive CD8^+^ T cell response(s). It should be noted that the JUNV GPC_18–26_ and MACV GPC_18–26_ epitopes have an identical amino acid sequence. The remaining 19 HLA-restricted arenavirus epitopes did not induce any detectable cross-reactive CD8^+^ T cell responses. Table 3 summarizes the results of these experiments.

**Table 3:** Summary of cross-reactive arenavirus peptides.

Arenavirus	Peptide^a^	Peptide sequence^b^	% of Epitope response^c^	Binding affinity to A*0201 or A*1101 (nM)^d^
JUNV	GPC_18–26_	ALNIALVAV	100	**79**
MACV	GPC_18–26_	---------	100	**79**
SABV	GPC_18–26_	-I----I--	23	834
WWAV	GPC_18–26_	------I--	74	**27**
LCMV	GPC_447–455_	YLVSIFLHL	100	**35**
LASV	GPC_441–449_	--I------	76	**5.5**
WWAV	GPC_428–436_	-VS------	20	**14**
MACV	NP_19–27_	GLSQFTHTV	100	**18**
JUNV	NP_19–27_	------Q--	24	**30**
MACV	NP_432–440_	AMPGVLSYV	100	**8.4**
GTOV	NP_432–440_	-Q--L----	50	**161**
JUNV	NP_432–440_	---------	100	**8.4**
SABV	NP_433–441_	-Q--L----	58	**114**
LCMV	GPC_46–55_	LVSFLLLAGR	100	**70**
LASV	GPC_46–55_	--T----C--	147	-
MACV	NP_82–90_	SIQKNTIFK	100	**5.5**
SABV	NP_82–90_	-S-RD--L-	47	**9.3**

a Position of epitope and cross-reactive peptide within prototypic strains of arenavirus GPC and NP.

b Full amino acid sequence is shown for epitope/immunizing peptide. Boldface letters indicate amino acid differences between epitope and cross-reactive arenavirus peptide.

c CD8^+^ T cells from peptide-immunized HLA-A*0201 and HLA-A*1101 transgenic mice were tested for their ability to produce IFN-γ in response to HLA-restricted target cells that had been pulsed with peptide. Percent of epitope response is calculated according to the formula: [(net spots/10^6^ CD8^+^ T cells against cross-reactive peptide)/(net spots/10^6^ CD8^+^ T cells against epitope)×100]. Data are representative of at least two independent experiments.

d Peptides were tested for binding to their respective HLA allele. Bold font highlights biologically relevant binders (IC_50_≤500 nM). −, Peptides with binding affinity >5000 nM. Binding affinities for LCMV GPC_447–455_, LASV GPC_441–449_, and WWAV GPC_428–436_ peptides are referenced from [10] and J. Botten and M. J. Buchmeier, unpublished work.

The amino acid sequence differences between the epitope sequence and the cross-reactive peptides mainly represented conservative or semi-conservative amino acid substitutions occurring at either the position 2 primary MHC interacting anchor residue or the prominent secondary anchor residues at positions 1, 3, and 7 (Table 3). Thus, in general, the residues involved in TCR interaction were preserved in the cross-reactive peptides. We also observed that non-cross-reactive peptides contained 3 or more amino acid differences with the epitope sequence. As detailed in Table 3, in terms of response magnitude, CD8^+^ T cell responses to the cross-reactive peptides ranged from 20–100% of the epitope-specific response.

**Figure 4:**
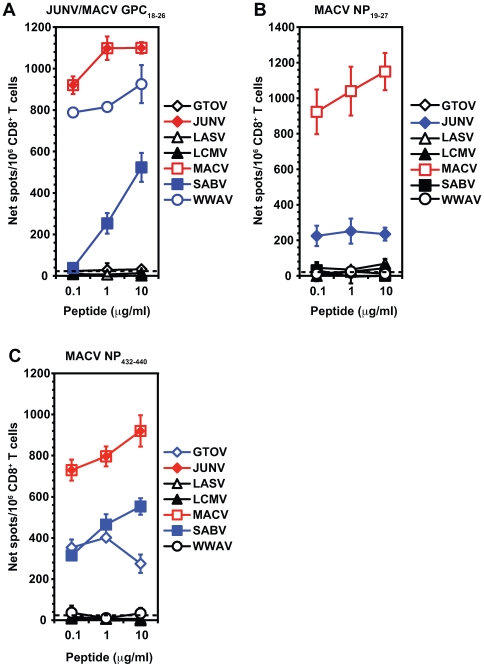
Immunization with arenavirus epitopes induces cross-reactive CD8^+^ T cell responses. HLA-A*0201 transgenic mice were immunized s.c. with either (A) JUNV/MACV GPC_18–26_, (B) MACV NP_19–27_, or (C) MACV NP_432–440_. Purified splenic CD8^+^ T cells were isolated 11 to 14 days later and exposed to JA2.1 cells that had been pulsed with 10, 1 or 0.1 µg/ml of the orthologous arenavirus peptides in an *ex vivo* IFN-γ ELISPOT assay. Red line indicates immunizing epitope, blue line indicates cross-reactive peptides, and black line indicates non-cross-reactive peptides. Responses were considered positive if the net SFC/10^6^ CD8^+^ T cells ≥20, the SI ≥2.0, and the statistical significance using a Student's *t* test had a p-value ≤0.05 at 10, 1 and 0.1 µg/ml of peptide in at least two independent experiments. The horizontal dashed line indicates the threshold for peptide positivity (net SFC/10^6^ cells ≥20). Error bars indicate SD. Results are representative of at least two independent experiments.

In most cases, the cross-reactivity detected was limited to New World arenaviruses, and in some cases, remained detectable even with low doses (0.1 µg/ml) of peptide (Figure 4). However, the LCMV GPC_447–455_ cross-reactivity spanned both Old (LASV) and New (WWAV) World arenaviruses (data not shown). A cross-reactive T cell response induced by LCMV GPC_447–455_ peptide immunization has also been reported in an independent study (J. Botten and M. J. Buchmeier, unpublished work). A total of 11 cross-reactive peptides, including 3 previously defined epitopes and 8 newly identified peptides, were recognized in repeat experiments by HLA-restricted epitope-specific CD8^+^ T cells. Summed together, 36 arenavirus-derived peptides, which include 28 epitopes and 8 cross-reactive peptides, generate arenavirus-specific CD8^+^ T cell responses that recognize the 7 different arenavirus species (Table 4). Thus, cross-reactive recognition of peptides significantly increases epitope coverage of the various HLA-virus combinations.

**Table 4:** Summary of HLA epitope coverage amongst 7 pathogenic arenaviruses.

HLA	GTOV	JUNV	LASV	LCMV	MACV	SABV	WWAV	Total
A*0201	1	4	4	5	3	4	4	25
A*1101	1	0	1	3	2	3	1	11
Total	2	4	5	8	5	7	5	36

Includes the sum of the arenavirus-derived epitopes and cross-reactive peptides defined in the present study, and the HLA-A*0201-restricted LASV and LCMV epitopes identified in previous studies [9],[10].

### Population coverage afforded by the arenavirus epitopes

As expected, the majority of peptides bound to their restricting allele with high affinity (IC_50_≤500 nM; Tables 1 and 2). Thus, our predictive approach effectively identified high affinity HLA binding peptides. As HLA-A02 and A03 supertypes are composed of a group of alleles that share largely overlapping peptide binding specificities [21], we subsequently tested the binding affinity of the identified arenavirus determinants to the remaining HLA molecules found within each supertype. All of the HLA-A*0201 determinants, except LASV GPC_111–120_, bound 3 or more additional HLA-A02 supertype molecules (HLA-A*0202, -A*0203, -A*0206, and -A*6802), and all HLA-A*1101 determinants bound 3 or more additional HLA-A03 supertype molecules (HLA-A*0301, -A*3001, -A*3101, -A*3301, and -A*6801) (data not shown).

Based on these results, we calculated the theoretical population coverage afforded by the epitopes and cross-reactive peptides for each of the 7 arenaviruses (using the Population Coverage Calculation Tool available through the Immune Epitope Database and Analysis Resource [22]). These calculations were based on peptide binding data (shown in Tables 1, 2, and 3 and data not shown) and the reported frequencies of each HLA allele in different ethnic populations. For the present analysis, biologically relevant binding was defined as an IC_50_≤500 nM.

**Figure 5:**
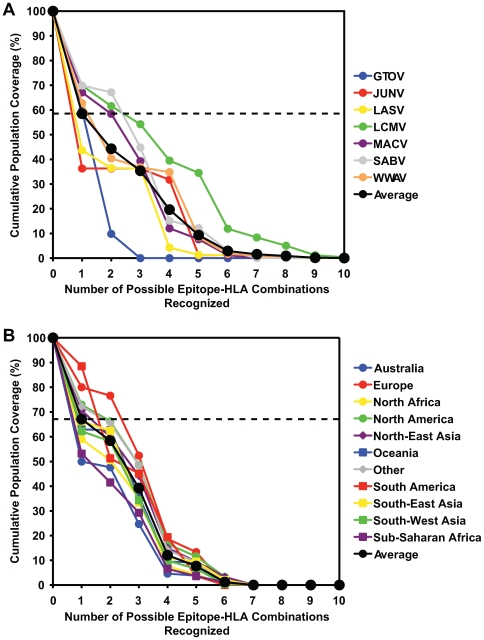
The identified epitopes and cross-reactive peptides provide broad population coverage. The theoretical population coverage was calculated based on the binding affinity data for each HLA-A02 and HLA-A03-restricted epitope and cross-reactive peptide as well as the reported frequencies of each HLA allele in different ethnic populations. A biologically relevant binding was defined as an IC_50_≤500 nM [19]. (A) The average population coverage for each of the 7 arenaviruses. The horizontal dashed line indicates that 58.5% of the population, on average, recognizes one or more arenavirus peptide. (B) For MACV, shown as representative data, the number of possible peptide-HLA allele combinations as a function of the fraction of each ethnic population (%) is shown. The horizontal dashed line represents the fraction of individuals (67.1%) that recognize one or more MACV peptide in an average population.

The total coverage of the general population provided by the corresponding set of epitopes for each of the 7 different arenaviruses is shown in Figure 5A. The epitopes identified from LCMV and SABV provided the broadest population coverage of 69.9% of the overall population, while JUNV epitopes provided the least amount of coverage of 36.3%. The remaining viruses, GTOV, LASV, MACV, and WWAV, provided 59.8%, 43.6%, 67.1%, and 62.7% population coverage, respectively. When averaged over the 7 different viruses, the defined epitopes provided population coverage of 58.5% of the entire population. In most cases, coverage entails recognition of multiple HLA-epitope combinations. For example, from Figure 5A, it can be seen that in an average population, about 35% of individuals can be expected to recognize 3 or more epitope-HLA combinations.

Coverage was fairly balanced throughout the major ethnic groups. A representative graph (Figure 5B) shows the coverage afforded by the MACV peptides across several major population groups. As shown, the MACV epitopes provide coverage ranging from about 50.0% of Australians, to 88.5% for South Americans, with an average coverage across all populations of 67.1%. These results indicate that the set of arenavirus epitopes described in the present study can provide broad coverage to the different viruses in different ethnic populations.

### Arenavirus epitope cocktail protects against rVACV challenge

Lastly, we examined whether priming of HLA-A*0201 transgenic mice with a pool of the HLA-A02 arenavirus peptides would confer protection against subsequent viral challenge. Because challenge with 6 of the 7 different arenavirus species considered would require BSL-4 containment, which was unavailable to us, we utilized challenge with rVACVs expressing the various arenavirus antigens as an alternative surrogate system.

**Figure 6:**
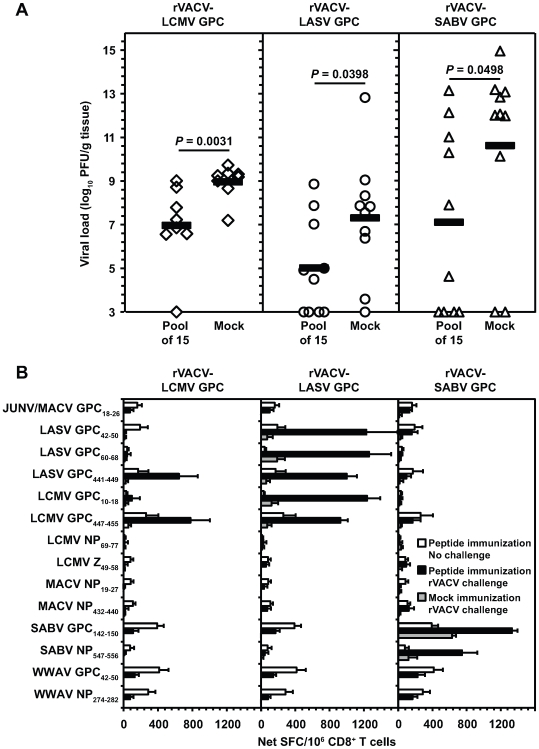
Protection against rVACV challenge is arenavirus epitope-specific in HLA-A*0201 transgenic mice. Female HLA-A*0201 transgenic mice were immunized with a pool of 15 peptides, including 14 arenavirus peptides plus the HBV core_128–140_ helper epitope, or with adjuvant and helper epitope alone (mock). On day 13 post-immunization, mice were inoculated i.p. with 10^7^ PFU of either rVACV-LCMV GPC, rVACV-LASV GPC, or rVACV-SABV GPC. (A) Ovaries were harvested 5 days after challenge, and viral titers were determined via plaque assay. Individual viral titers are shown and the horizontal line represents the mean titer for each group. When no plaque growth was detected, values were set at a threshold of detection (log_10_ pfu/g tissue = 3.0). Mean viral titers from the peptide-immunized group were compared to the mock group using the Mann-Whitney *t* test to determine if differences were significant. (B) On day 18 post-immunization, splenic CD8^+^ T cells were isolated from mice that were immunized with peptide alone, immunized with peptide and challenged with a rVACV 5 days previous to the assay, or immunized with adjuvant and challenged with a rVACV. CD8^+^ T cells were stimulated with peptide (0.1 µg/ml) pulsed JA2.1 cells in an *ex vivo* IFN-γ ELISPOT assay. Error bars indicate SD. Results are an average of two independent experiments.

Groups of HLA-A*0201 transgenic mice were immunized with a pool of 14 CD8^+^ T cell peptides plus a single T helper cell epitope unrelated to arenaviruses, or mock-immunized (see Materials and Methods for details). To ensure protection was HLA-restricted, arenavirus peptides that were also restricted by murine MHC (LASV_111–120_ and LCMV GPC_34–43_) and not endogenously processed in human APCs (JUNV GPC_7–15_) were excluded. Peptide-immunized mice were challenged with a rVACV construct that expressed either LCMV GPC, LASV GPC, or SABV GPC. On day 5 post-challenge, we observed that peptide pool-immunized mice had significantly reduced mean viral titers following challenge compared to the mock controls (Figure 6A) with rVACV-LCMV GPC (2.0 log_10_ reduction; *P* = 0.0031), rVACV-LASV GPC (2.3 log_10_ reduction; *P* = 0.0398), and rVACV-SABV GPC (3.5 log_10_ reduction; *P* = 0.0498).

Although the results obtained from mice challenged with rVACV-LCMV GPC were quite consistent, we observed varying viral titers in both mock and peptide-immunized mice challenged with either rVACV-LASV GPC or rVACV-SABV GPC. This could be due to technical failure in delivering the proper infectious dose of these rVACV constructs during the i.p. inoculation. Despite this variability, there still remains a significant difference (*P*<0.05) between viral titers of the mock and peptide-immunized mice that were challenged with rVACVs expressing either LASV GPC or SABV GPC. We also found that the 3 different groups of mock-immunized mice had varying viral titer levels. All rVACVs utilized are capable of high titer replication both *in vitro* and *in vivo*. We have, however, observed that certain rVACVs (i.e. rVACV-SABV GPC) grow more efficiently compared to others (i.e. rVACV-LCMV GPC). This might help explain the differences observed in viral titers in mock-immunized mice challenged with the 3 rVACVs (Figure 6A).

To determine whether an expansion of epitope-specific CD8^+^ T cells after rVACV challenge would correlate with viral titer reduction, the frequency of epitope-specific CD8^+^ T cells following peptide pool immunization before and after rVACV challenge was measured. At 18 days post-peptide pool immunization, HLA-A*0201 transgenic mice that were not challenged displayed significant numbers of CD8^+^ T cells to all immunizing epitopes (Figure 6B). However, mice that were peptide pool immunized and challenged with an rVACV-GPC demonstrated a clear expansion of CD8^+^ T cells specific for the challenge virus. In some instances, cross-reactive CD8^+^ T cell expansion was also observed. For example, LASV GPC_441–449_-specific CD8^+^ T cells expanded following challenge with rVACV-LCMV GPC. Thus, these results demonstrate that CD8^+^ T cell-mediated immunity can protect against challenge with rVACVs expressing Old and New World arenavirus antigens in an epitope-specific manner.

## Discussion

Hemorrhagic fever and aseptic meningitis caused by arenavirus infection represent serious human public health problems that lead to dehabilitating and sometimes fatal disease. Furthermore, arenaviruses are regarded as a potential bioterrorism threat, and as such are classified as Class A pathogens. Development of a vaccine against arenavirus infection needs to address the genetic diversity observed within and between different viral species of the *Arenaviridae* family. Indeed, pathogen heterogeneity is also a prominent consideration among other families of RNA viruses. For instance, the genetic variability found amongst HIV clades circulating worldwide, and the high mutation rate of HIV that allows for evasion of the adaptive immune response, continue to provide a daunting challenge for vaccine development [23]. Likewise, HCV vaccine design faces the obstacle of different genotypes that are found with different prevalence in distinct locations [24]. The four dengue virus serotypes provide a unique challenge, as heterologous re-infection is associated with dengue hemorrhagic fever and dengue shock syndrome [25],[26]. The variability associated with influenza virus is reflected in different strains and subtypes, forcing the development of yearly updated vaccines, and is also the cause of concern in the context of new influenza pandemics [27],[28].

As it is unlikely that separate vaccines for each viral species within a family will be developed, the two most viable, and not necessarily exclusive, approaches might be the development of either a multivalent and/or a cross-protective vaccine. Herein, we performed proof of concept studies to evaluate these approaches, utilizing arenaviruses as a model system. Our results indicate that multiple conserved (89 to 100%) epitopes can be defined from each arenavirus. While broadly cross-reactive epitopes between arenavirus species were not identified, occasional cross-reactivites were demonstrated, leading to an increased viral coverage by the defined epitope set. Notably, it was possible to induce simultaneous responses against all epitopes by a peptide pool vaccination, demonstrating the feasibility of a multivalent vaccination. This shows that a single vaccination strategy with both multivalent and cross-protective CD8^+^ T cell epitopes was able to engender protection from recombinant viruses expressing antigens derived from a subset of different arenavirus species. Because our study was focused on demonstrating universal coverage against multiple arenavirus species, we chose to conduct protection studies utilizing rVACVs expressing antigens from 3 representative arenavirus species. Protection from virus challenge was demonstrated through reduced viral titers in peptide-immunized animals instead of survival, as the rVACVs used in this study are attenuated viruses. Previous studies utilizing animal models of HIV and influenza infection have demonstrated that a reduction in viremia correlates with survival [29],[30]. Importantly, we have also shown in an independent study that immunization with LCMV GPC_447–455_ protected HLA-A*0201 transgenic mice against lethal intracranial challenge with LCMV [31].

To identify HLA-restricted CD8^+^ T cell epitopes derived from pathogenic arenaviruses, certain technical challenges needed to be circumvented, including the need for BSL-4 containment for most species considered, and the lack of availability of samples from exposed human donors. We bypassed the requirement for BSL-4 containment by developing a panel of 24 rVACV vectors that expressed the different arenavirus antigens of interest. To our knowledge, this is the first instance in which this approach has been implemented on a large scale to study T cell responses directed against human pathogens. In addition, utilizing HLA transgenic mice enabled the identification of epitopes relevant for humans. HLA transgenic mice have been useful in identifying human T cell epitopes from small viruses, such as influenza [32], and more complex pathogens, such as VACV [33]. In the future, we plan to examine CD8^+^ T cell responses in arenavirus-immune human donors in order to assess the degree of overlap between arenavirus-specific responses recognized in HLA transgenic mice and humans.

In total, 16 HLA-A*0201, and 10 HLA-A*1101, arenavirus-specific CD8^+^ T cell epitopes that are naturally processed by human APCs have been identified so far. These numbers are similar to those found in other virus infection models. For example, we previously identified 14 HLA-A*0201, 4 HLA-A*1101, and 3 HLA-B*0702-restricted VACV-specific CD8^+^ T cell epitopes using HLA transgenic mice [34]. In additional studies in H-2^b^ mice, 19 H-2K^b^ and 9 H-2D^b^-restricted LCMV-specific epitopes were identified [8]. In the VACV system, 27 H-2K^b^ and 22 H-D^b^-restricted epitopes were defined [35]. It is important to point out that our current study was not designed to identify the totality of the response, but rather focused on epitopes relatively well conserved within a given arenavirus species.

Widely conserved CD8^+^ T cell epitopes among the 7 different arenaviruses species were not identified. Given that amino acid sequence identities of homologous proteins of the 7 arenavirus species range from 44 to 63% [36], it is not surprising that only a few conserved epitopes were identified. However, we found that immunization of HLA transgenic mice with 7 of the arenavirus peptides induced cross-reactive CD8^+^ T cell responses, which significantly increased the coverage afforded by the epitope set against different arenavirus species. Importantly, T cell cross-reactivity might also extend to newly identified arenaviruses. To examine this further, we compared the amino acid sequence of the epitopes defined here to the orthologous regions within Chapare [37] and Lujo [38] viruses. We found that 6 of the identified epitopes had ≥70% amino acid sequence identity to the Chapare and Lujo viruses. Thus, cross-reactive T cells might also extend to peptides derived from newly discovered arenaviruses.

Previous studies have demonstrated T cell cross-reactivity for both Old and New World arenaviruses. It was shown in a guinea pig model that adoptive transfer of LCMV or Moepia virus (MOPV)-immune CD8^+^ T cells could confer protection against challenge with the highly virulent LASV [39]. Likewise, in a murine model, cell transfer of LCMV-immune CD4^+^ and CD8^+^ T cells protected mice from Pichinde virus infection [40]. Furthermore, in humans, T cell clones from patients that have recovered from acute LASV infection showed cross-reactive recognition of MOPV peptides [41]. Inter-clade cross-reactivity has also been demonstrated for HIV-specific CD8^+^ T cell responses and is considered of particular relevance in vaccines that are to be used in geographically and genetically distinct HIV epidemics [42],[43],[44].

We demonstrated that the arenavirus epitope set afforded redundant coverage of the different viruses by epitopes of different restriction, as an average about 50% of individuals are predicted to recognize more than one MHC-peptide combinations. Coverage of approximately 60% of the general population is projected on the basis of HLA frequencies and binding data. As only 2 HLA supertypes were investigated, it should be possible to achieve essentially 100% coverage by identifying epitopes restricted by additional supertypes, such as A01, A24, B07, and B44 [16],[21].

Herein, we implemented a multivalent vaccine strategy to protect against rVACVs expressing antigens from different arenavirus species. This approach has proven itself in the case of the currently licensed vaccine against HPV, which contains capsid proteins derived from 4 different serotypes prominently associated with disease [45]. We demonstrated that immunization of HLA-A*0201 transgenic mice with a cocktail containing 14 arenavirus-derived peptides and a T helper cell epitope resulted in a detectable CD8^+^ T cell response to each of the viral epitopes. The magnitude of epitope-specific CD8^+^ T cell responses following peptide pool immunization was diminished compared to the responses observed after individual peptide immunization (see Figures 2 and 6B for comparison). Competition for HLA-A*0201 binding might play a role in determining the response magnitude of epitopes when immunized as a pool. However, the magnitude of the CD8^+^ T cell responses was sufficient to significantly reduce viral titers following challenge with rVACVs expressing both Old and New World arenavirus antigens.

Furthermore, comparing our data to a previous study, we found that the coverage achieved with the breadth of epitopes does not compromise immunity to a single epitope. HLA-A*0201 transgenic mice immunized with the pool of the 14 arenavirus-specific determinants and challenged with rVACV-LASV GPC demonstrated a 2.3-log reduction in viral titers compared to the mock control. Botten *et al.* demonstrated that single peptide immunization of HLA-A*0201 transgenic mice with either LASV GPC_42–50_ or GPC_60–68_, two epitopes included in our peptide pool immunization, followed by challenge with rVACV-LASV GPC resulted in a 2.8 and 2.2-log reduction in viral titers, respectively [9]. Thus, viral titer reduction is very similar whether mice are immunized with a single epitope or an epitope cocktail.

None of the defined arenavirus epitopes demonstrated cross-reactivity with VACV-WR, strongly suggesting that the reduction in viral titers in peptide-immunized mice is mediated by arenavirus-specific CD8^+^ T cell responses (see Figure 1 for representative data). To provide further evidence for arenavirus epitope-specific protection, we observed a substantial expansion of CD8^+^ T cell responses specific for the challenge virus (relative to unchallenged mice). Furthermore, Botten *et al.* demonstrated that immunization of HLA-A*0201 transgenic mice with either LASV GPC_42–50_ or GPC_60–68_ did not result in protection against challenge with the irrelevant rVACV-LASV NP construct, confirming the protection following rVACV-LASV GPC challenge was LASV epitope-specific [9].

We also found cross-reactive CD8^+^ T cell responses were significantly boosted following rVACV challenge of peptide pool-immunized mice. As expected, the LCMV GPC_447–455_-specific CD8^+^ T cell response was boosted following rVACV-LASV GPC challenge, while challenge with rVACV-LCMV GPC boosted the CD8^+^ T cell response against LASV GPC_441–449_. The LCMV GPC_10–18_-specific CD8^+^ T cell response expanded approximately 40-fold following rVACV-LASV GPC challenge, while only 3-fold after challenge with rVACV-LCMV GPC. One possible explanation for this discrepancy is that the expanded CD8^+^ T cell subset generated following rVACV-LASV GPC challenge has a higher avidity for the LCMV peptide. Finally, mice immunized with the peptide pool and challenged with the rVACV expressing SABV GPC showed a significant expansion of CD8^+^ T cells with the SABV NP_547–556_ peptide. It is plausible that infection with rVACV-SABV GPC led to the generation of a cross-reactive CD8^+^ T cell subset that was capable of recognizing the SABV NP_547–556_ peptide.

In conclusion, our studies suggest that simultaneous induction of a multivalent and cross-protective CD8^+^ T cell response is a feasible approach to vaccination against multiple arenavirus species. As a proof of concept, protection studies with rVACVs expressing the GPC from LCMV, LASV, and SABV were performed. Additional studies are still required to investigate whether peptide pool immunization would reduce viral titers in transgenic mice challenged with rVACVs expressing other arenavirus GPCs (GTOV, JUNV, MACV, and WWAV), or other arenavirus proteins (NP and/or Z). The identification of epitopes restricted by additional HLA types, their validation in humans exposed to arenavirus infections, and their formulation in a multivalent construct would also be the logical next steps in the further exploration of this concept.

## Materials and Methods

### Ethics statement

All mouse studies followed guidelines set by the National Institutes of Health and the Institutional Animal Care and Use Committee-approved animal protocols (Association for Assessment and Accreditation of Laboratory Animal Care International (AAALAC) and Office of Laboratory Animal Welfare (OLAW)).

### Bioinformatic analyses

The arenavirus open reading frames (ORFs) utilized in this study were a total of 333 unique sequences from one or more isolates of GTOV, JUNV, LASV, LCMV, MACV, SABV, and WWAV taken from the arenavirus protein sequence database (http://epitope.liai.org:8080/projects/arena) [36]. Candidate HLA-A02 and A03 supertype epitopes were identified using a previously described algorithm [15]. Peptides with a predicted IC_50_≤100 nM were selected from each ORF, and then ranked based on inter-virus conservancy (% identity matches). The top ranked peptides (up to 30) per supertype per virus per antigen were selected.

### Peptide synthesis

Peptides were synthesized as crude material by Pepscan Systems (Lelystad, The Netherlands). Candidate epitopes were resynthesized by A and A Labs (San Diego, CA) and purified to 95% or greater homogeneity by reverse-phase HPLC. Hepatitis C virus core 132 (DLMGYIPLV), and human MAGE 369 (SSLPTTMNY) were used as control HLA-A*0201 and HLA-A*1101-restricted peptides, respectively. IEDB submission identification numbers for HLA-A*0201 and HLA-A*1101-restricted epitopes are 1000381 and 1000374, respectively.

### MHC peptide-binding assay

Quantitative assays to measure the binding affinity of peptides to purified HLA-A02 (A*0201, A*0202, A*0203, A*0206, A*6802) and HLA-A03 (A*1101, A*0301, A*3001, A*3101, A*3301, A*6801) supertype molecules were based on the inhibition of binding of a radiolabeled standard peptide, and were performed essentially as described elsewhere [46],[47],[48]. Briefly, after a 2-day incubation, binding of the radiolabeled peptide to the corresponding MHC class I molecule was determined by capturing MHC/peptide complexes on Greiner Lumitrac 600 microplates (Greiner Bio-One, Monroe, NC) coated with the W6/32 Ab (anti-HLA class I), and measuring bound cpm using the Topcount microscintillation counter (Packard Instrument). The concentration of peptide yielding 50% inhibition of the binding of the radiolabeled probe peptide (IC_50_) was then calculated. Peptides were typically tested at six different concentrations covering a 100,000-fold dose range, and in three or more independent assays. Under the conditions utilized, where [label] < [MHC] and IC_50_ ≥ [MHC], the measured IC_50_ values are reasonable approximations of the K_D_ values.

### Mice

HLA-A*0201/K^b^ and HLA-A*1101/K^b^ (referred to as HLA-A*0201 and HLA-A*1101) transgenic mice were bred and maintained in the animal facilities at the La Jolla Institute for Allergy and Immunology (La Jolla, CA). These mice were the F_1_ generation derived from a cross between BALB/c mice (The Jackson Laboratory, Bar Harbor, ME) and HLA transgenic mice (expressing a chimeric gene consisting of the α1 and α2 domains of the human HLA and the α3 domains of murine H-2K^b^) created on the C57BL/6J background [17],[18]. CB6F1/J mice were the F_1_ generation derived from a cross between C57BL/6J and BALB/c mice (The Jackson Laboratory). C57BL/6J mice deficient in K^b^ and D^b^ (K^b^D^b−/−^) mice were purchased from Taconic Farms (Hudson, NY).

### Cells

Target cells used in the IFN-γ ELISPOT assays were the human Jurkat cells (JA2.1) that express the HLA-A*0201/K^b^ chimeric gene [17], human EBV B cells (BVR) that express HLA-A*1101, and LPS-stimulated B lymphoblasts prepared as previously described [34] from HLA-A*0201 and HLA-A*1101 transgenic mice, and CB6F1/J (H-2^bxd^) mice, which do not express the HLA transgene. The target cells were either pulsed for 1 h with peptide (10, 1, or 0.1 µg/ml), or infected with 10^7^ PFU rVACV, or 2×10^6^ PFU VACV-WR 24 h prior to the ELISPOT assay. CV-1 (CCL-70), Vero E6 (CRL-1586), BHK-21 (CCL-10), and BSC-40 (CRL-2761) cells were purchased from ATCC and grown as recommended by the supplier.

### Viruses

The rVACV-LCMV GPC and NP (derived from LCMV Armstrong Clone 53b) constructs were engineered using the WR strain of VACV as previously described [49]. LCMV GPC and NP gene expression were under the control of the VACV P7.5K early/late promoter [49]. The remaining rVACV arenavirus constructs were also generated on the WR background using ORF sequences from prototypic arenavirus strains (as outlined in Table S1), according to the protocols established by Blasco and Moss [50]. Arenavirus target gene expression was under the control of a synthetic early/late promoter, PSYN [50]. In brief, the arenavirus GPC, NP, L, and Z genes were subcloned individually into the pRB21 transfer vector. CV-1 cells were then infected with the VACV strain vRB12 at an MOI of 2, followed by transfection with 10 µg of the transfer vector containing a single arenavirus ORF. Viruses that underwent homologous recombination with the transfer vector were selected for their plaque-forming ability. Arenavirus protein expression was confirmed for each rVACV through immunoflourescence assay to verify that all plaques expressed the correct antigen, as well as by Western blot analysis to verify the correct processing and size of each arenavirus protein. Virus titers were determined through plaque assays on BSC-40 cells.

### Immunizations and viral challenge

For epitope identification studies, HLA-A*0201 and HLA-A*1101 transgenic mice were peptide-immunized s.c. at the base of the tail with a pool of 8 to 11 peptides (15 µg/peptide) or 50 µg of a single peptide, along with 140 µg of the helper I-A^b^-restricted epitope hepatitis B virus (HBV) core_128–140_ (TPPAYRPPNAPIL) in PBS emulsified in IFA. In general, effector CD8^+^ responses were analyzed in the spleens of mice 11 to 14 days after peptide immunization. One exception was the assessment of processing of the HLA-A*1101 peptides. In this case, effector CD8^+^ responses were analyzed by utilizing splenocytes from single peptide-immunized mice that were cultured *in vitro* for an additional 6 days with the immunizing peptide. For VACV infection, HLA-A*0201 transgenic mice were injected i.p. with either 2×10^6^ PFU VACV-WR strain as a control, or 10^7^ PFU rVACV containing one of the arenavirus genes. A higher dose of 10^7^ PFU rVACVs was used because these viruses are attenuated, while wt VACV-WR is not. The infectious doses of 2×10^6^ PFU VACV-WR and 10^7^ PFU rVACV have been routinely used in previous studies [9],[31],[34]. On day 7 post-infection, mice were sacrificed and purified splenic CD8^+^ T cells were analyzed by *ex vivo* ELISPOT assays for IFN-γ.

For challenge studies, HLA-A*0201 transgenic mice immunized with a pool of 15 peptides, including 14 arenavirus epitopes (50 µg/peptide) and 140 µg of the HBV core_128–140_ helper epitope, in PBS emulsified in IFA, or mock-immunized with an equivalent volume of DMSO as the peptide-immunized group as well as 140 µg of the helper epitope in PBS emulsified in IFA. Mice were challenged 13 days later through i.p. inoculation with 10^7^ PFU rVACV-LCMV GPC, rVACV-LASV GPC, or rVACV-SABV GPC. Five days after virus challenge, spleens were harvested for CD8^+^ T cells and ovaries were harvested for rVACV titer determination. To quantitate viral titers, ovaries were homogenized and sonicated prior to plating serial 10-fold dilutions of the homogenates on BSC-40 cells.

### IFN-γ ELISPOT assay

The mouse IFN-γ ELISPOT assay was performed as previously described [51]. In brief, 4×10^5^ splenocytes or 2×10^5^ splenic CD8^+^ T cells (purified by anti-CD8 magnetic beads [Miltenyi Biotec, Auburn, CA]) were cultured with either 10^4^ or 10^5^ peptide-pulsed or rVACV-infected target cells. For peptide pulsing, target cells were incubated with peptide for at least 1 h at 37°C, followed by 3 washes to remove excess peptide. For rVACV infection, JA2.1 or BVR cells were infected at a MOI of 10 with a rVACV 18 to 20 h prior to the assay. Each assay was performed in triplicate wells. After a 20 h incubation at 37°C, plates were developed, and responses calculated as described [34]. Criteria for positivity were net spot-forming cells (SFC)/10^6^ cells ≥20, stimulation index (SI) ≥1.4 or 2.0, and p-value ≤0.05 using a Student's *t* test in at least 2 out of 3 experiments.

## Supporting Information

Figure S1 Expression of GTOV antigens from rVACV constructs. BSC-40 cells were infected with rVACV encoding the GTOV NP, GPC, L, or Z proteins or wt VACV and 24 hr later protein lysates were generated from infected cells. Protein lysates were run on a 4–20% tris-glycine acrylamide gel, transferred onto a nitrocellulose membrane, and probed via Western blot with an anti-HA antibody to detect the presence of GTOV NP, GPC/GP2, L, and Z (all of which contained a C-terminal HA tag). Two protein bands are detected in the rVACV-GTOV GPC cell lysate; the upper band is GPC and the lower band is GP2, the post-translational cleavage product of GPC. The size of the molecular weight markers in kiloDaltons is indicated on the left-hand side of the blot.
(0.36 MB PDF)

Table S1 Arenavirus strain sequences used to generate rVACV constructs expressing the GPC, L, NP, or Z protein.
(0.05 MB PDF)
