# Supplementary figures and images for: A Multivalent and Cross-Protective Vaccine Strategy against Arenaviruses Associated with Human Disease
Source: PLoS Pathog. 2009 Dec 18;5(12):e1000695. doi: 10.1371/journal.ppat.1000695 (PMC2787016; doi:10.1371/journal.ppat.1000695)

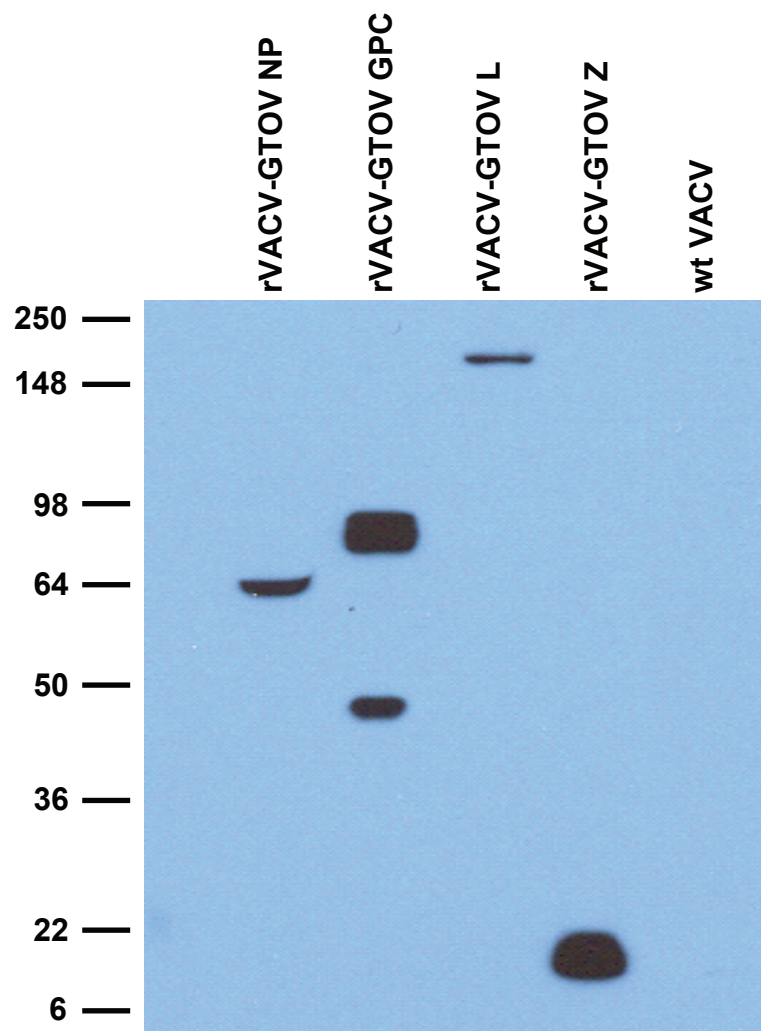

Supplement: Figure S1 — Expression of GTOV antigens from rVACV constructs. BSC-40 cells were infected with rVACV encoding the GTOV NP, GPC, L, or Z proteins or wt VACV and 24 hr later protein lysates were generated from infected cells. Protein lysates were run on a 4–20% tris-glycine acrylamide gel, transferred onto a nitrocellulose membrane, and probed via Western blot with an anti-HA antibody to detect the presence of GTOV NP, GPC/GP2, L, and Z (all of which contained a C-terminal HA tag). Two protein bands are detected in the rVACV-GTOV GPC cell lysate; the upper band is GPC and the lower band is GP2, the post-translational cleavage product of GPC. The size of the molecular weight markers in kiloDaltons is indicated on the left-hand side of the blot. (0.36 MB PDF) [file ppat.1000695.s001.pdf]
