# Supplementary material for: A Multivalent and Cross-Protective Vaccine Strategy against Arenaviruses Associated with Human Disease
Source: PLoS Pathog. 2009 Dec 18;5(12):e1000695. doi: 10.1371/journal.ppat.1000695 (PMC2787016; doi:10.1371/journal.ppat.1000695)
Supplement: Table S1 — Arenavirus strain sequences used to generate rVACV constructs expressing the GPC, L, NP, or Z protein. (0.05 MB PDF) [file ppat.1000695.s002.pdf]

Table S1. Arenavirus strain sequences used to generate rVACV constructs expressing the GPC, L, NP, or Z protein.

Arenavirus	Strain	Gene	Gene Identifier	Protein Locus
GTOV	INH-95551	GPC	22901285	AAN09938
		L	33868617	AAQ55254
		NP	23307849	AAN05424
		Z	33868616	AAQ55253
JUNV	Candid #1 (for GPC & NP)	GPC	33868613	AAQ55251
		L	33868611	AAQ55250
	XJ cl13 (for L)	NP	33868614	AAQ55252
LASV	Josiah	GPC	23343511	NP_694870
		L	37787710	AAT49002
		NP	23343510	NP_694869
		Z	23343513	NP_694871
LCMV	Armstrong 53b	GPC	23334589	NP_694851
		L	67126	AAA66591
		NP	331360	AAA46257
		Z	331386	AAA46268
MACV	Carvallo	GPC	22901291	AAN09942
		L	33868608	AAQ55248
		NP	22901292	AAN09943
		Z	34365536	NP_899214
SABV	SPH114202	GPC	1480446	AAC55091
		NP	1480447	AAC55092
		Z	33868627	AAQ55262
WWAV	AV 9310135	GPC	14333983	AAK60497
		NP	14333984	AAK60498
